# Evaluation of anticancer effect of colchicum autumnale L. Corm on breast cancer cell

**DOI:** 10.1186/s12906-023-04189-0

**Published:** 2023-10-07

**Authors:** Shiva Falahianshafiei, Javad Akhtari, Ali Davoodi, Hajar Pasha

**Affiliations:** 1 Department of Pharmacy, Ayatollah Amoli Branch, Islamic Azad University, Amol, Iran; 2 Department of Medical Nanotechnology, School of Advanced Technologies in Medicine, Mazandaran University of Medical Sciences, Sari, Iran; 3 Department of Pharmacognosy and Biotechnology, Mazandaran University of Medical Sciences, Sari, Iran; 4 Social Determinants of Health Research Center, Health Research Institute, Babol University of Medical Sciences, Babol, Iran

**Keywords:** Breast cancer, Cytotoxicity, MCF-7, MTT, Colchicum autumnale plant, Doxorubicin

## Abstract

**Background:**

Breast cancer is the most common malignancy in women, and medicinal plants can prevent and play an inhibitory role for cancer. This study aims to evaluate the anticancer effect of colchicum autumnale L. Corm on breast cancer cell models.

**Methods:**

In this study, the alkaloid-rich extract was prepared using the percolation method and with methanol/water solvent (70:30). HFF2 normal cell line and MCF-7 breast cancer cell line were cultured in microplates (96 wells). Then cells were treated with concentrations of 62.5 to 2000 ng/ml of extract and concentrations of 62 to 1000 ng/ml of doxorubicin at regular intervals of 48 and 72 h, and the percentage of cell growth inhibition was calculated. Cytotoxicity of drugs was measured by the MTT assay method. IC50 values were calculated by Calcusyn software. Also, the *P*-value of < 0.05 was considered significant.

**Results:**

Alkaloid-rich extract of Colchicum autumnale plant inhibited breast cancer cell growth (MCF-7). The IC50 parameter showed more cytotoxic effects of Colchicum autumnale plant extract on the MCF-7 cancer cell line than HFF2 normal cell line for 48 and 72 h. In addition, with higher concentrations of the extract, cytotoxicity, and growth inhibitory effect increased significantly and in comparison to the doxorubicin was almost the same as cytotoxic.

**Conclusion:**

This research provides a novel view into the development of new drugs for the treatment of cancer diseases. Colchicum autumnale plant extract had a significant cytotoxic effect like Doxorubicin drug on breast cancer cell line (MCF-7), which can alternatively treat and prevent breast cancer.

## Introduction

As a chronic and non-communicable disease, it comprises a wide group of diseases and is considered a major health problem affecting the health of society [1]. Cancer is a preliminary global health concern, and researchers strive for innovations to combat the disease [2]. According to the reports of the World Health Organization, the number of deaths caused by cancer has increased significantly in the world [3]. In Iran the highest prevalence can be found in breast, stomach, colorectal, and esophageal cancers [4]. Breast cancer is the most common malignancy in women worldwide and the second most common cause of death from cancer. A number of environmental and genetic risk factors are at interplay such as age, obesity, family history of breast cancer [5], socioeconomic status, body mass index, age at menarche and menopause, use of hormone therapy, consuming alcohol, and smoking for the occurrence of cancer [6]. Regarding breast cancer, the cells which originate in the breast tissue begin to multiply irregularly and exponentially [7].

Cancer can be treated by surgery, radiation therapy, and chemotherapy agents. Multitudes of cancer chemotherapy agents have been synthesized, but they have many unpleasant side effects [8]. Currently, high costs, efficiency constraints, and adverse side effects have discouraged patience from compliance with drug protocols [9]. Therefore, there is urgency for new models in the design of potential chemotherapeutic agents, and natural products provide such models [8].

Medicinal plants are propounded as effective therapeutic agents, and in vitro studies have identified novel drug candidates from plant extracts [2]. Today, there has been an imperative to develop new, effective anticancer agents to overcome resistance and reduce severe side effects [9], and encourage people to rely more heavily on traditional medicine. Medicinal plants have an almost unlimited capacity to produce substances and can help researchers for new chemotherapy agents [8]. Traditionally plants have been harvested for their medicinal properties to treat ailments. Herbal medicines have been regarded as efficient agents to prevent and treat cancer, and medicinal plants are commonly available and economically affordable [10]. The compounds extracted from medicinal plants have a major role in the prevention and treatment, and in many cases, these bioactive compounds can be applied for the design of effective and potent drugs with fewer side effects and are more efficacious than primary molecules [11, 12]. Herbal medicine has natural compounds that interact harmoniously with the body, while synthetic treatments are associated with a range of adverse effects, from mild to severe, and might harm the organs [2, 13].

One of the flowering and perennial plant families that have been widely used in traditional medicine is the Colchicaceae flower family. One of the important genera of this family and the only genera found in Iran is the flower of Hasrat or Suranjan plant [14, 15]. Colchicum autumnale plant has tropolone alkaloid compounds. Due to having an anti-cell proliferation effect, these compounds are a suitable option to prevent cancer cell proliferation [16, 17]. The main active ingredient of this genus is an alkaloid called colchicine. Among the different types of flowers of Hasrat, Suranjan is the best source of Colchicine [18]. Colchicine can effectively bind to tubulin and inhibit the polymerization of microtubules. Based on earlier studies, colchicine is an excellent anti-mitotic substance like vinca alkaloids and has anticarcinogenic and antiproliferative properties in different cell lines and animal models [19, 20].

Tropolone alkaloids such as colchicine are the main bioactive compounds of Colchicum kind and have anti-inflammatory effects with potent anti-cancer properties through tubulin polymerization inhibitory mechanisms [21, 22]. Such compounds could be applied to design the most effective colchicine anti-tubulin in compounds with better pharmacodynamics and pharmacokinetics parameters [23]. Michigan Cancer Foundation 7 (MCF-7) is a breast cancer cell line and a suitable model for breast cancer investigation. This cell is Estrogen receptor/progesterone receptor (ER/PR) positive and belongs to the luminal molecular subgroup [24].

More research is required in the treatment of malignancies through medicinal plants as they have various therapeutic properties, due to the presence of the high prevalence of malignant diseases and the side effects of chemotherapy drugs which inflict a high cost on the families. In this study we aimed to investigate the anticancer effect of Colchicum autumnale L. Corm on the breast cancer cell model and compared it with the Doxorubicin drug.

## Methods

### Plant materials

Colchicum autumnale L. tuber samples were purchased from Niak Pharmaceutical Company, Gorgan, Iran. In addition to, Dr. Masoud Azadbakht done taxonomic identification of plant samples, and confirmed. Representative voucher specimens were deposited in the herbarium of the Department of Pharmacognosy in Faculty of Pharmacy of the Mazandaran University of Medical Science, Sari, Iran. Furthermore, herbarium number was defined BE1-11314. Additionally, one of the researchers involved in this study is a pharmacognosy specialist who has conducted similar studies in this field [23].

After fine powdering of the plant up to 200 mesh, extraction was done by percolation method and with methanol/water solvent (70:30) under conditions of 10 times the solvent. Then the obtained extracts were concentrated under a vacuum and dried with a freeze-dryer [25, 26].

### The cultivation of cell lines

The MCF-7 cell line (breast cancer cells) in this study was obtained from the Iranian Cell and Molecular Biology Research Center (CMBRC; Tehran, Iran). Both cells were cultured in Dulbecco’s Modified Eagle Medium (DMEM) supplemented with fetal bovine serum (FBS) at a concentration of 10 percent in microplates (96 wells). Gibco and other reputable companies were the sources of all the materials necessary for cell culture. All other solvents and reagents were used as a chemical grade.

In order to culture cells and prepare their growth conditions, cells were cultured in T25 flasks and frozen for later use, when not needed and placed in -80 freezers or liquid nitrogen tanks. In the initial cryopreservation of cells, the stock cells were removed from their storage place (liquid nitrogen tank). After that, they were allowed a short time under the hood to leave the frozen state. Then some of them were poured into the tube and about 5 ml of complete culture medium (RPMI culture medium, Sigma, Germany, containing 10% fetal bovine serum) was added to it. It was centrifuged at 1500 rpm for 5 minutes. After the cells were collected at the bottom of the tube, the supernatant, which contains dimethyl sulfoxide (DMSO) and is toxic to the cells, was discarded.

The washing process was repeated. Then, a few milliliters of complete culture medium was added to the cell mass, and by pipetting with the tip of the blue sampler, it became a uniform cell suspension. This cell suspension was transferred to a cell culture flask and a complete culture medium was added to it to form a layer of culture medium at the bottom of the flask. The flask was transferred to a 37 °C incubator containing 5% CO2.

In order to pass the cells, within a few days after cell culture in flask 25, the culture medium was changed every day so that the substances resulting from cell metabolism were removed from the vicinity of the cells. After this period, the cells were fully grown and cell masses (without a microscope) were visible at the bottom of the flask. Then, the flask of grown cells was transferred from the 37 °C incubator to the laminar hood, and the culture medium was completely removed from the flask. Trypsin–EDTA solution was applied to dissociate cells from one another. The contents of the flask were transferred to a tube and centrifuged at 1500 rpm for 5 minutes.

Cells were counted with a hemocytometer slide in 4 large squares. After obtaining the mean of the counted cells, the number of cells per milliliter of cell suspension was calculated. In all tests, the percentage of live cells was determined with trypan blue dye. Ten microliters of cell suspension was mixed with 90 microliters of trypan blue dye. In this case, the dilution factor was equal to 10.

### Cytotoxicity assay

Cytotoxicity effect was measured by the MTT assay method which is commonly used to check the toxicity of substances and formulations. This toxicity can be used on unhealthy cell lines such as cancer cells to study drugs or on healthy cells to ensure the safety of compounds.

First, the solution was prepared from the MTT powder. Then cells were cultured in 96 well plates. Throughout this study, cell culture was conducted in the growth and proliferation phase. First, one hundred microliters of cell suspension equivalent to five thousand cells per milliliter was poured into the wells of 96-well plates in each stage of the experiment. For about 24 hours, the plates were placed in a 37 °C incubator so that the cells would adapt to the environment and adhere to the bottom of the wells. There were a total of two compounds of colchicine extract and doxorubicin.

### Growth inhibition using MTT assay

To prepare the dilution from the extract and the positive control (doxorubicin), 5 dilutions were used for each formulation and 4 repetitions were considered for each dilution. The negative control consisted of 4 to 10 wells with cells and the culture medium. It also included all the substances in the test wells except for the drugs. For colchicine, 10 milligrams per milliliter of the prepared formulation was used. Serial dilutions of the formula and drug were prepared in sterile 2 ml microtubes and added to the wells of the 96-well plate containing cells with four repetitions. The plates were placed in a 37-degree incubator for 48 and 72 hours. After the end of the drug and cell incubation period, MTT solution with a concentration of 5 mg/ml of PBS was added to each well. The plates were placed in a 37-degree incubator again for 4 hours. At the end of the culture medium, the wells were slowly drained and formazan dye crystals in the cytoplasm of the cells were completely dissolved by adding 100-200 microliters of DMSO using an 8-channel sampler for each well. Color intensity (light absorption intensity) was determined with an ELISA reader at a wavelength of 490-570 nm. All steps were repeated four times and the average light absorbance was taken as the mitochondrial activity.

### Quality control measures

For quality control measures during the experiments, the results of cell culture obtained from three experiments are completely independent, and different tests have been carried out to adjust the concentrations. The range of concentrations for colchicine extract was from 62 to 2000 ng/ml and for doxorubicin it was from 62 to 1000 ng/ml. The concentrations used in the MTT assay were obtained by examining a wide range of colchicine concentrations on cells and selecting the optimal concentration range. Regarding the known drug doxorubicin, the researchers relied on the results of earlier studies and their own experience, although this drug has been used as a control in this experiment. The results of examining the variables were recorded in the form of graphs in measuring devices (ELISA device or MTT test) and then this information related to the effect of drugs on the cell growth was presented with the IC50 index (a concentration of the drug that reduces cell growth by 50% compared to the control). IC50 values were calculated by Calcusyn software.

### Data analysis

The data of this study were analyzed using statistical T-tests (comparing the mean IC50, and concentrations of drugs) and the SPSS V.22 statistical program. The significance level for all tests was considered less than 0.05.

## Results

The amount of total alkaloid tropolone in the extract as prepared based on colchicine was 0.92 ± 0.05 mg/kg. IC50 value of plant extract and control drug in normal and cancer cells after 48 and 72 h was calculated using Calcusyn software (Table 1).

**Table 1:** IC50 value of plant extract and control drug in normal and cancer cells after 48 and 72 h

IC50 value	Colchicum autumnale extract		Doxorubicin drug		*P*-Value
	**48 hours**	**72 hours**	**48 hours**	**72 hours**	
**Normal cells (ng/ml)**^a^	223.54	137.72	134.56	83.64	.288
**Cancer cells (ng/ml)**	129.95	87.43	127.43	85.92	.952

^a^nanograms per milliliter

As shown in Table 1, the concentrations of 223.54 ng/ml and 137.72 ng/ml of colchicum autumnale extract and 134.56 ng/ml and 83.64 of doxorubicin drug inhibited more than 50% of normal cells after 48, and 72 h, respectively. There was no significant difference in the mean of IC50 in normal cells between doxorubicin and colchicum autumnale extract (109.10 ± 36.01 vs 180.63 ± 60.68, *P* = 0.288; respectively). Furthermore; the concentrations of 129.95 ng/ml and 87.43 ng/ml of colchicum autumnale extract and 127.43 ng/ml and 85.92 ng/ml of doxorubicin drug inhibited 50% of cancer cells after 48, and 72 h, respectively. Also no significant difference was found in the mean of IC50 in cancer cells between doxorubicin and colchicum autumnale extract (106.67 ± 29.35 vs 108.69 ± 30.07, *p* = 0.952; respectively). The growth inhibition effect of the extract in both times was as effective as with doxorubicin. However, the lowest IC50 value in cancer cells and normal cells was related to Colchicum autumnale extract than the control drug after 48 and 72 h.

**Fig. 1:**
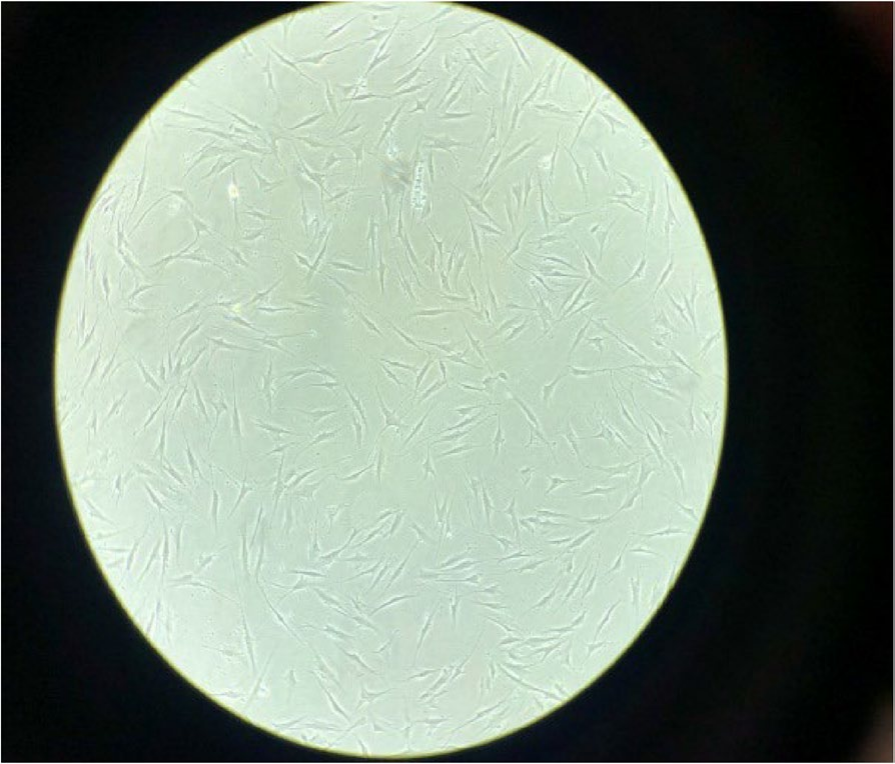
HFF-2 cells

**Fig. 2:**
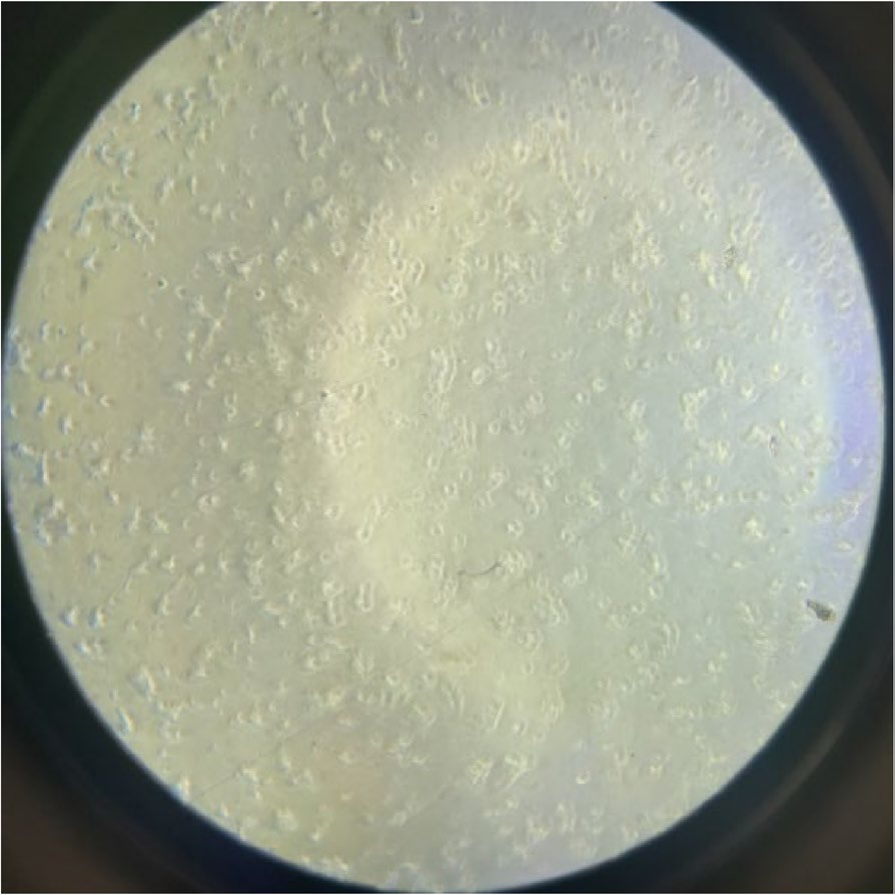
MCF-7

**Fig. 3:**
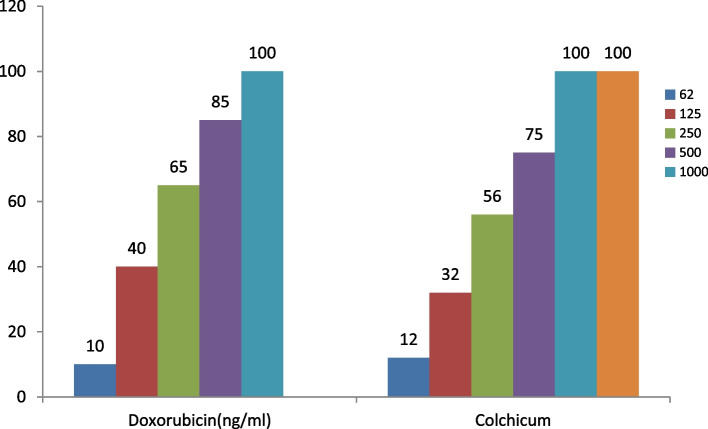
The growth inhibition effect of Doxorubicin and Colchicum autumnale plant extract on the HFF-2 cell line in different concentrations after 48 h

**Fig. 4:**
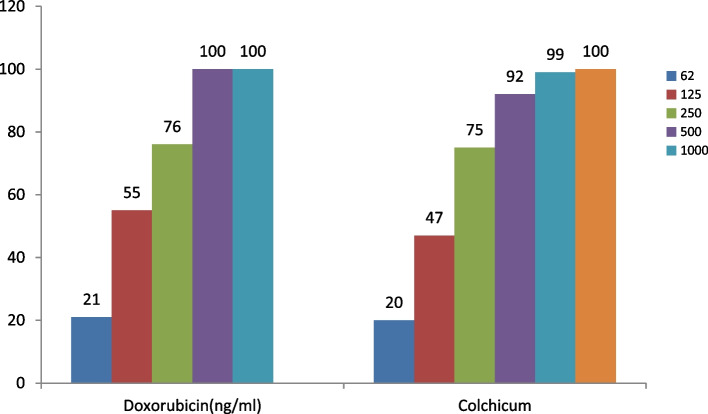
The growth inhibition effect of Doxorubicin and Colchicum autumnale plant extract on the HFF-2 cell line in different concentrations after 72 h

**Fig. 5:**
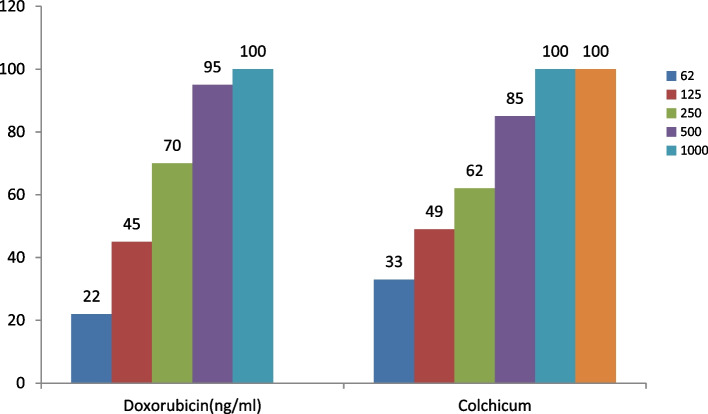
The growth inhibition effect of Doxorubicin and colchicum autumnale plant extract on MCF-7 cell line in different concentrations after 48 h

**Fig. 6:**
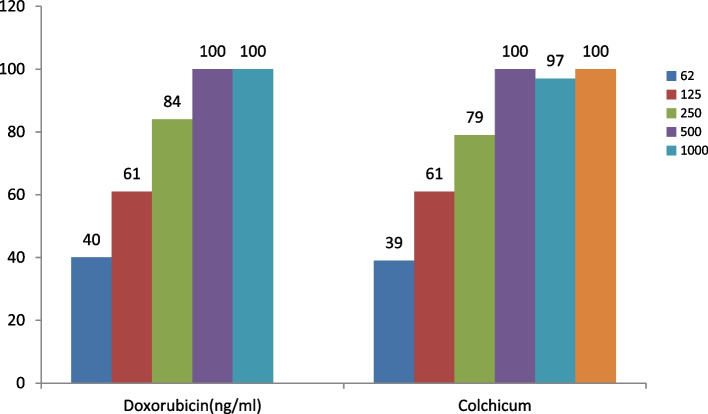
The growth inhibition effect of Doxorubicin and Colchicum autumnale plant extract on MCF-7 cell line in different concentrations after 72 h

In this study, the effectiveness of doxorubicin and the alkaloid-rich extract of colchicum autumnale L. on two normal (HFF-2) (Fig. 1) and cancerous cell lines (MCF-7) (Fig. 2) after 48 and 72 h were quantitatively estimated, as shown in Figs. 3, 4, 5 and 6.

Figure 3 shows the effectiveness of doxorubicin and colchicum autumnale plant extract on the HFF-2 normal cell line in concentrations of 62, 125, 250,500, 1000 ng/ml, and 62, 125, 250, 500, 1000, 2000 ng/ml after 48 h, respectively. The percentage of normal cell growth inhibition increases with higher concentration of colchicum autumnale extract so in the concentration of 62 ng/ml of extract, we have the lowest percentage of growth inhibition and at the concentration of 1000 and 2000 ng/ml of extract with the highest percentage of growth inhibition. The toxicity effect of both compounds was dose-dependent and there was a direct relationship between the concentration and the growth inhibition effect. There was no significant difference in the mean of growth inhibition between doxorubicin and colchicum autumnale extract on the HFF-2 cell line in different concentrations after 48 h (60.00 ± 35.88 vs 55.00 ± 33.65, *p* = 0.828; respectively).

Figure 4 reveals the effectiveness of doxorubicin and colchicum autumnale plant extract on the normal HFF-2 cell line in concentrations of 62, 125, 250, 500, and 1000 ng/ml, and 62, 125, 250, 500, 1000 and 2000 ng/ml after 72 h, respectively.

The percentage of normal cell growth inhibition rose with greater concentration of colchicum autumnale extract (at the concentration of 62 ng/milliliter of extract). The toxicity effect of both compounds was dependent on the dosage and there was a direct relationship between the concentration and the growth inhibition effect. There was no significant difference in the mean of growth inhibition between doxorubicin and colchicum autumnale extract on the HFF-2 cell line in different concentrations after 72 h (70.40 ± 33.39 vs 66.60 ± 32.87, *p* = 0.861; respectively).

Figure 5 presents the effectiveness of doxorubicin and colchicum autumnale plant extract on the MCF-7 cell line in concentrations of 62, 125, 250, 500, and 1000 ng/ml, and 62, 125, 250, 500, 1000 and 2000 ng/ml after 48 h, respectively. There was a higher percentage of cancer cell growth inhibition at higher concentrations of colchicum autumnale extract so in the concentration of 62.5 ng/ml of extract. The toxicity effect of both compounds was dose-dependent and there was a direct relationship between the concentration and the growth inhibition effect. In addition no significant difference was detected in the mean of growth inhibition between doxorubicin and colchicum autumnale extract on the MCF-7 cell line in different concentrations after 48 h (66.40 ± 33.13 vs 26.97 ± 12.06, *p* = 0.976; respectively).

Our data indicate the effectiveness of doxorubicin and colchicum autumnale plant extract on the MCF-7 cell line in concentrations of 62, 125, 250, 500, and 1000 ng/ml, and 62.5, 125, 250, 500, 1000 and 2000 ng/ml after 72 h, respectively. The growth inhibition of cancer cells increased in personage at greater concentrations of colchicum autumnale extract so that at 62.5 ng/ml of extract, there was the lowest percentage of growth inhibition and at 500 and 2000 ng/ml of extract, the highest percentage of growth inhibition was found. The toxicity effect of both compounds was dose-dependent and there was a direct relationship between the concentration and the growth inhibition effect. Moreover, no significant difference was found in the mean of growth inhibition between doxorubicin and colchicum autumnale extract on the MCF-7 cell line in different concentrations after 72 h (77.00 ± 26.13 vs 75.20 ± 25.58, *p* = 0.915; respectively). The effect of growth inhibition on this normal cell appeared in higher concentrations compared to cancer cells, and with increasing exposure time, the inhibitory concentration decreased (Fig. 6).

## Discussion

In this study, the impact of a cytotoxic extract rich in alkaloids of the colchicum autumnale plant was evaluated on two normal cell lines (HFF2) and a cancer cell line (MCF-7) after 48 and 72 h, and then compared to the doxorubicin control drug.

Our data demonstrated that Alkaloid-rich extract of Colchicum autumnale plant inhibited breast cancer cell growth (MCF-7). Several previous studies have also evaluated the impact of cytotoxic effects of Colchicine. For instance, a survey by Adham Foumani et al. (2022) revealed that the isolated colchicine from Colchicum autumnale, similar to standard colchicine, modulated the expression levels of several genes and inhibited cell proliferation. Therefore colchicine can be a potential candidate for prevention and treatment of breast cancer [20]. Additionally, an in vitro and ex vivo a study by Urbaniak et al. (2019) to investigate Carbamate derivatives of Colchicine show potent activity toward primary acute lymphoblastic leukemia and primary breast cancer cells, and reported that Colchicine and two of its most active derivatives could terminate human breast cancer cells, stopped mitosis in MCF-7 cells, and triggered Microtubule depolymerizing [27]. In another study, in evaluating colchicinoids from Colchicum crocifolium bioss (Colchicaceae) was identified a new colchicinoid analogue from extracts of the whole plant as N, N-dimethyl-N-deacetyl-(-)-cornigerine. All compounds revealed potent cytotoxicity effect against MCF-7 human breast cancer, H460 human large cell lung cancer, and SF-268 human astrocytoma cell lines [28].

Our findings revealed that the alkaloid-rich extract of colchicum autumnale plant in different concentrations from 62 to 2000 ng/ml on the MCF-7 cell line after 48 and 72 h had strong cytotoxic effects. This effect was more evident in higher concentrations, i.e. 1000 and 2000 ng/ml. Moreover, the cytotoxic effects of the alkaloid-rich extract of colchicum autumnale plant in higher concentrations (1000 and 2000) after 48 h and concentrations greater than 500 (500, 1000, and 2000) after 72 h were almost similar. The cytotoxic effect of colchicum autumnale extract gradually increased with greater concentrations. The toxicity effect of both compounds was dose-dependent and there was a direct relationship between the concentration and the growth inhibition effect. In line with this study, Adham Foumani et.al (2022) showed that Colchicine of colchicum autumnale induced apoptosis in a dose-dependent manner significantly in cancer cell lines (MCF-7) [20]. Furthermore, Shirzad et al. (2020) revealed that with increasing concentration of essential oil, cytotoxicity was augmented significantly [29]. Also, Bakar-Ateş et al. (2018) studied the effect of colchicine with concentrations of 0.1, 10, and 100 μg/ml in stopping the cell cycle and mRNA expression related to Matrix metalloproteinase-2 (MMP-2) on MCF-7 breast adenocarcinoma cells and found that colchicine at concentrations of 10 and 100 μg/ml significantly prevented cell viability. It inhibited the cell cycle in the G2/M phase and decreased the expression of mRNA related to MMP-2 from MCF-7 cells at all therapeutic concentrations [30]. Previous research has shown that the rate of early apoptosis, and delayed and necrosis in the treated cell lines increased compared to the control, and the expression of the BCL2 gene in the cell lines treated with a concentration of 1.25 mg/ml of plant extract for 24 and 48 h was significantly reduced compared to the beta-actin control gene. It has been concluded that the hydroalcoholic extract of Blepharis persica seeds can decelerate the proliferation of breast and prostate cancer cells and induce apoptosis in cancer cells, although in low concentration it does not enhance the anticancer effects [31]. Another study in 2015 showed that colchicine produced significant dose-dependent anti proliferative effects in both human bile duct cancer cell lines (C14 / KMUH and C51 / KMUH). Experiments on naked mice have shown that an increase in the ratio of tumor volume in mice treated with colchicine was significantly lower than that in control mice using clinically acceptable plasma concentrations of colchicine [32].

The result of the present study provided evidence of a high level of cellular toxicity in both colchicum autumnale extract and the doxorubicin on MCF-7 cancer cells after 72 h compared to 48 h. Maximum percentage of cell toxicity occurred with longer duration. There was an increase in cellular toxicity of both colchicum autumnale extract and doxorubicin after 72 h than that of 48 h on normal HFF-2 cells, which emphasizes that with longer durations, the effect of extract and medicine is augmented. A review of the literature showed that colchicine (with different concentrations of 0, 0.01, 0.1, and 1 μm) inhibited the growth and proliferation of hypopharyngeal cancer cells in different times (after 24, 48, and 72 h) and dose-dependent manner [33]. Sun et al. in 2016 evaluated the inhibiting effect of colchicine 24, 48, and 72 h on the proliferation and apoptosis of MCF-7 breast cancer cells and showed that the inhibitory effect of colchicine gradually increased with longer exposure periods and concentration [34].

Studies have demonstrated that growth-inhibition colchicum autumnale extract against MCF-7 cancer cells was approximately equal with doxorubicin control. On the other hand, the colchicum autumnale extract was effective on MCF-7 cancer cells, and in comparison to the doxorubicin it is almost the same as cytotoxic. There was no significant difference in the mean of growth inhibition between doxorubicin and colchicum autumnale extract on the MCF-7 cell line in different concentrations after 48 and 72 h. In addition, no significant difference was found in the mean of IC50 in Normal cells between doxorubicin and colchicum autumnale extract. In this regard, Agha Abbasi et al. (2018) reported that the hydroalcoholic extract of Blepharis persica seeds had the greatest growth inhibition effect on prostate, breast, and fibroblast cell lines, respectively. The combined effect of doxorubicin with the extract in all three cell lines was not significantly different from the control [31]. Furthermore the same study revealed that Colchicinoid's effect on growth inhibition against MCF-7 cancer cells was approximately equal to with positive control, camptothecin [35], While Shirzad et al. (2020) revealed that the cytotoxic effect of S. Setifera plant essential oil on MCF-7 human breast cancer cells was significantly higher compared to the control group. The essential oil had a severe cytotoxicity compared to the drug doxorubicin [29].

According to the study’s results, the amount of IC50 extract for the cancer cell was less than the normal cell after 48 and 72 h, indicating that the extract and doxorubicin drug are more toxic to the cancer cells than the normal cell. On the other hand, extract toxicity was actually higher for cancer cells than normal cells, which was due to higher and uncontrollable proliferation of cancer cells. Ezzati Ghadi et al. (2020) showed that the hydroalcoholic extract of Colutea persica leaf had cytotoxicity and the viability percentage of breast cancer cells was lower than that of fibroblasts. Also, the rate of early apoptosis, delayed, and necrosis in the treated cell lines increased compared to the untreated ones [35]. In this regard, it should note the extract's safety profile and potential clinical. The processing of natural agents can be associated with toxicity concerns, and ensuring that the appropriate dosage is necessary to guarantee safety. In this connection, different carriers are used and connected to different compounds. They are also employed like liposomes to reduce their toxic effects or change their bioavailability and biodistribution. Furthermore, the results of a previous review from this study conducted by Azadbakht et al. (2020) on the effect of alkaloid toxicity from Colchicum and Colchicaceae showed low hemolytic activity and cytotoxicity [23]. However, through precise usage of herbal medicine, more effective and less toxic pharmaceuticals are likely to be developed than those solely relying on refined medications [13].

### Limitations

A major limitation of using this protocol is the challenges involved in the preparation and high cost associated with these materials. It is suggested to investigate the cytotoxic effects of the extract in other concentrations and with other control drugs. More studies in animal models and investigation of the toxicity of the extract in vivo models are suggested.

## Conclusion

This paper has been noted that Colchicum autumnale plant extract has anticancer activity against breast cancer. Also, the cytotoxic effect of the alkaloid-rich extract of Colchicum autumnale plant on the MCF-7 cancer cell line was almost equal to the control drug, doxorubicin. Based on these results, we suggest that Colchicum autumnale can be considered a potential candidate for the treatment and prevention of breast cancer. Therefore, it can be used as an anti-breast cancer drug in the laboratory stage. However, in-vitro results require to be approved by in vivo studies. Therefore, we have planned to conduct other experiments such as apoptosis assay and preclinical studies in animal models.

## Data Availability

The data supported during the present research are available from the corresponding author upon reasonable request.
